# Leukocyte Count and Adverse Clinical Outcomes in Acute Ischemic Stroke Patients

**DOI:** 10.3389/fneur.2019.01240

**Published:** 2019-11-26

**Authors:** Kehua Quan, Anxin Wang, Xiaoli Zhang, Yongjun Wang

**Affiliations:** ^1^Department of Neurology, Beijing Tiantan Hospital, Capital Medical University, Beijing, China; ^2^China National Clinical Research Center for Neurological Diseases, Beijing, China; ^3^Center of Stroke, Beijing Institute for Brain Disorders, Beijing, China; ^4^Beijing Key Laboratory of Translational Medicine for Cerebrovascular Disease, Beijing, China

**Keywords:** ischemic stroke, leukocyte count, recurrent stroke, all-cause death, functional outcomes, inflammation

## Abstract

**Background:** Post-ischemic inflammatory response might be affected by many factors. We chose leukocyte count as a marker of inflammatory response and investigated whether the effects of leukocyte count on the clinical outcomes in acute ischemic stroke patients are different according to different factors.

**Methods:** We derived data from the China National Stroke Registry II. Patients with ischemic stroke were classified into four groups by leukocyte count quartiles within the first 24 h after admission. Adverse clinical outcomes were defined as recurrent stroke, all-cause death, and poor functional outcomes (3 ≤ mRS ≤ 5) at 3-months and 1-year follow-up. The subgroup factors were age, sex, history of hypertension, history of diabetes, history of previous stroke, or transient ischemic attack and smoking status. We assessed the association between leukocyte count and adverse clinical outcomes and evaluated this association in different subgroups.

**Results:** A total of 14,678 patients were included. Patients in higher quartiles were likely to be younger, male, smokers, and drinkers, and to have a shorter time from symptom onset to arrival, a more proportion of history of diabetes, atrial fibrillation, and hypertension, and a higher severity of stroke. Higher quartiles were associated with elevated risk of adverse clinical outcomes at 3-months and 1-year follow-up. Leukocyte count had a moderate accuracy to predict clinical outcomes. There was no difference in the relationship between leukocyte count and adverse clinical outcomes across subgroups such as age, sex, history of hypertension, and smoking. The effect of leukocyte count on all-cause death was pronounced among patients with previous stroke or transient ischemic attack, and the effect of leukocyte count on short-term poor functional outcomes was also pronounced among patients without diabetes.

**Conclusions:** Leukocyte count is associated with short-term and long-term clinical outcomes of acute ischemic stroke and may have predictive value, especially in patients with certain specific characteristics.

## Introduction

The inflammatory response has been considered to be associated with all stages of ischemic stroke and results in the development of ischemic injury and the exacerbation of neurological function (1–3). Leukocyte count as a marker of inflammation, on the one hand, is connected with the severity of ischemic damage (4, 5). Early leukocytosis is found to be related to the volume of infarcted tissue among acute ischemic stroke patients (6). On the other hand, previous studies have suggested that leukocyte count is a significant independent predictor of poor clinical outcomes and discharge disability (5). It is also connected with an increased risk of all-cause mortality after ischemic stroke (7). Moreover, increased leukocyte count is reportedly associated with a higher risk of recurrent ischemic stroke (8).

Post-ischemic inflammatory response is a complicated process that might be affected by many factors such as age, sex, and so on. Some of those factors are characterized by a chronic low-grade inflammatory state. Those factors may influence acute ischemic stroke outcomes through inflammatory response. For example, emerging data demonstrate differences in the composition of circulating and infiltrating leukocytes recruited to the ischemic brain of old male mice after stroke compared to young male mice (9). Besides, an exacerbated inflammatory response to acute ischemic stroke in old mice leads to more severe brain damage and behavioral dysfunction (10). Recent studies suggest that sex differences in the immune response to ischemic stroke may also contribute to outcomes (11). It is reported that in the experimental mouse model of diabetes, acute inflammatory responses are perturbed in the brain following stroke, and the alteration is associated with the exacerbation of stroke-induced injury (12). Moreover, an animal experiment observes that pre-existing hypertension causes larger stroke sizes possibly as consequence of a profound increase of post-stroke inflammation (13).

Yet, those factor-dependent roles of inflammatory response associated with increased ischemic brain injury have not been fully elucidated. Whether the results obtained from animal experiments could be translatable to ischemic stroke patients is also uncertain now. In addition, as far as we know, there are limited studies focused on effects of inflammatory response on the ischemic stroke outcomes under different factors at present. So, we chose leukocyte count as a marker of post-ischemic inflammatory response and conducted this study in order to investigate whether the effects of leukocyte count on the prognosis in patients with acute ischemic stroke are different in different factor subgroups.

## Methods

### Study Population

We derived data from the China National Stroke Registry II (CSNR II). The CSNR II is a nationwide, multicenter, prospective registry study launched by the Ministry of Health of China aiming to establish a reliable national stroke database and evaluate the delivery of stroke care in clinical practice (14). A total of 25,018 patients were enrolled consecutively from 219 hospitals voluntarily participating in the CSNR II from June 2012 to January 2013 that met the following criteria: (1) age >18 years; (2) diagnosis within 7 days of the index event of ischemic stroke, transient ischemic attack, spontaneous intracerebral hemorrhage, or subarachnoid hemorrhage confirmed by brain imaging; (3) direct hospital admission from a physician's clinic or emergency department; and (4) informed consent provided by the patient or a legally authorized representative. The protocol of the CNSR II study was approved by the Central Institutional Review Board of the Beijing Tiantan Hospital. All patients or their representatives provided written informed consents before participation.

Among all the enrolled patients in the CSNR II, 19,604 were diagnosed with ischemic stroke. After the exclusion of patients with in-hospital pneumonia (*n* = 1,501), missing available leukocyte count (*n* = 1,315), and lost to follow-up (*n* = 2,110), 14,678 patients were included in our analysis.

### Data Collection

Baseline information, including age, sex, smoking and drinking status, medical history, and time from symptom onset to arrival, was systematically collected through face-to-face interviews during hospitalization by trained research coordinators at each participating hospital. The National Institutes of Health Stroke Scale (NIHSS) score at admission was assessed by the trained neurologists blinded to patients' clinical information.

### Leukocyte Count Testing

Fasting whole blood samples from venipuncture were taken within the first 24 h after admission into a vacutainer tube containing EDTA and kept at room temperature. Afterwards, leukocyte count was analyzed by automated hematology analyzer within 1 h after sample collection at each research center. All measurements were performed by laboratory personnel blinded to subjects' clinical situations.

### Outcome Assessment

Patients enrolled were followed up by telephone interview at 3 months and 1 year after stroke onset according to the protocol of CSNR II study. Endpoint events, including recurrence of stroke, all-cause death, and disability assessed by the modified Rankin Scale (mRS) score, were collected by trained research coordinators at each participating hospital who followed standard scripts and were blinded to patients' baseline characteristics. Adverse clinical outcomes were defined as recurrent stroke, all-cause death, and poor functional outcomes (3 ≤ mRS ≤ 5). Recurrent stroke included both ischemic and hemorrhagic stroke during follow-up period.

### Statistical Analysis

We presented continuous variables as median with interquartile range and categorical variables as proportions. Patients enrolled were classified into four groups by leukocyte count quartiles. Baseline data were compared across leukocyte count quadruplet groups using Fisher's exact test for categorical variables and Kruskal–Wallis test for continuous variables. We used multivariate logistic regression to investigate the association between leukocyte count and adverse clinical outcomes. To adjust for other potential confounding variables, multivariable analyses including age, sex, hypertension, hyperlipidemia, diabetes, previous stroke or transient ischemic attack, myocardial infarction, atrial fibrillation, drinking, smoking, and the NIHSS score at admission were performed. By the receiver operating characteristic (ROC) curves, we determined the accuracy of leukocyte count levels to serve as a prognosis. The area under the curve (AUC) was calculated as a criterion for the accuracy of the test. In the subgroup analysis, we chose age, sex, history of hypertension, history of diabetes, history of previous stroke or transient ischemic attack, and smoking status as subgroup factors. Firstly, we also used multivariate logistic regression to investigate the association of leukocyte count with adverse clinical outcomes in each subgroup and adjusted for the same potential confounding variables above. Besides, we performed a test for the interaction between leukocyte count and each subgroup factor. *P* < 0.05 for two-sided hypothesis testing was considered as statistically significant. All statistical analyses were conducted with SAS software version 9.4 (SAS Institute Inc., Cary, NC).

## Results

### Baseline Characteristics

A total of 14,678 patients were included in our analysis. Table 1 showed the baseline characteristics compared between the included and excluded patients. The median (IQR) leukocyte count was 6.695 (5.500–8.200) × 10^9^/L. Baseline characteristics of the included patients stratified into quartiles according to leukocyte count are shown in Table 2. Of all the patients, the median (IQR) age was 65.0 years (56.0–74.0), 5,364 (36.54%) were female, and the median (IQR) time from symptom onset to arrival was 23 h (6–56). Diabetes, atrial fibrillation, and hypertension were more frequently found in patients with higher quartiles and other medical histories were not different across quartiles. Median NIHSS scores at admission from the lowest to the highest quartile were 3 (2–6), 3 (2–6), 4 (2–6), and 4 (2–8), respectively. So, patients in higher quartiles were more likely to have severe neurologic deficit. Moreover, patients in higher quartiles were more likely to be with smoking and drinking history. In summary, compared with patients with lower leukocyte count, those in higher quartiles were more likely to be younger, male, smokers, and drinkers, and to have a shorter time from symptom onset to arrival, a more proportion of history of diabetes, atrial fibrillation, and hypertension, and a higher severity of stroke (Table 2).

**Table 1:** Baseline characteristics of included and excluded patients.

**Characteristics**	**Included patients** ***n* = 14,678**	**Excluded patients** ***n* = 4,926**	***P*-value**
**Demography and clinical features**			
Age, median (IQR), years	65 (56–74)	67 (58–76)	<0.0001
Female, *n* (%)	5,364 (36.54)	1,803 (36.60)	0.9425
Ever smoking, *n* (%)	6,451 (43.95)	2,221 (45.09)	0.1644
Ever drinking, *n* (%)	4,101 (27.94)	1,758 (35.69)	<0.0001
**Medical history**, ***n*** **(%)**			
Previous stroke/transient ischemic attack	4,875 (33.21)	1,765 (35.83)	0.0008
Diabetes	2,954 (20.13)	1,106 (22.45)	0.0005
Myocardial infarction	340 (2.32)	139 (2.82)	0.0468
Atrial fibrillation	924 (6.30)	458 (9.30)	<0.0001
Other coronary heart disease	1,680 (11.45)	614 (12.46)	0.0543
Hypertension	9,410 (64.11)	3,287 (66.73)	0.0009
Hyperlipidemia	1,661 (11.32)	709 (14.39)	<0.0001
Peripheral vascular disease	592 (4.03)	209 (4.24)	0.5203
NIHSS at admission, median (IQR)	4 (2–6)	4 (2–9)	<0.0001
Time from symptom onset to arrival, median (IQR), h	23 (6–56)	18 (4–50)	<0.0001

**Table 2:** Baseline characteristics of patients stratified by leukocyte count quartiles.

**Characteristics**	**Total**	**Leukocyte count quartiles**				***P*-value**
		**Q1**	**Q2**	**Q3**	**Q4**	
		***n* = 3,639**	***n* = 3,700**	***n* = 3,664**	***n* = 3,675**	
**Demography and clinical features**						
Age, median (IQR), years	65.0 (56.0–74.0)	67.0 (58.0–75.0)	65.0 (57.0–73.0)	64.0 (56.0–73.0)	63.0 (54.0–73.0)	<0.0001
Female, *n* (%)	5,364 (36.54)	1,510 (41.49)	1,356 (36.65)	1,217 (33.22)	1,281 (34.86)	<0.0001
Ever smoking, *n* (%)	6,451 (43.95)	1,399 (38.44)	1,571 (42.46)	1748 (47.71)	1,733 (47.16)	<0.0001
Ever drinking, *n* (%)	4,101 (27.94)	871 (23.94)	1,020 (27.57)	1,122 (30.62)	1,088 (29.61)	<0.0001
**Medical history**, ***n*** **(%)**						
Previous stroke/transient ischemic attack	4,875 (33.21)	1,163 (31.96)	1,206 (32.59)	1,242 (33.90)	1,264 (34.39)	0.0978
Diabetes	2,954 (20.13)	587 (16.13)	734 (19.84)	802 (21.89)	831 (22.61)	<0.0001
Myocardial infarction	340 (2.32)	76 (2.09)	89 (2.41)	83 (2.27)	92 (2.50)	0.6650
Atrial fibrillation	924 (6.30)	214 (5.88)	196 (5.30)	223 (6.09)	291 (7.92)	<0.0001
Other coronary heart disease	1,680 (11.45)	413 (11.35)	391 (10.57)	445 (12.15)	431 (11.73)	0.1788
Hypertension	9,410 (64.11)	2,219 (60.98)	2,389 (64.57)	2,408 (65.72)	2,394 (65.14)	<0.0001
Hyperlipidemia	1,661 (11.32)	384 (10.55)	427 (11.54)	434 (11.84)	416 (11.32)	0.3446
Peripheral vascular disease	592 (4.03)	131 (3.60)	156 (4.22)	155 (4.23)	150 (4.08)	0.4797
NIHSS at admission, median (IQR)	4 (2–6)	3 (2–6)	3 (2–6)	4 (2–6)	4 (2–8)	<0.0001
Time from symptom onset to arrival, median (IQR), h	23 (6–56)	24 (7–60)	24 (8–62)	23 (6–57)	17 (5–48)	<0.0001

### Association of Leukocyte Count With Adverse Clinical Outcomes

At 3-months follow-up, 595 (4.08%) patients had recurrent stroke, 507 (3.45%) patients died, and 2,136 (14.55%) patients had poor functional outcomes, while at 1-year follow-up, 847 (6.05%) recurrent stroke occurred, 1,032 (7.03%) patients ended up with death, and there are 1,859 (12.67%) patients with poor functional outcomes. The risk of adverse clinical outcomes in leukocyte count quartiles is shown in Table 3. Both at 3-months and 1-year follow-up, compared with the lowest quartile taken as reference, higher quartiles were significantly related to elevated risk of adverse clinical outcomes. That was to say the patients in higher quartiles were more likely to have adverse clinical outcomes. After adjustments for age, sex, hypertension, hyperlipidemia, diabetes, previous stroke or transient ischemic attack, myocardial infarction, atrial fibrillation, drinking, smoking, and the NIHSS score at admission, the association between quartiles of leukocyte count and adverse clinical outcomes was still significant. So, we considered that the leukocyte count of patients could influence clinical outcomes (Table 3).

**Table 3:** Risk of adverse clinical outcomes stratified by leukocyte count quartiles.

	**Q1**	**Q2**	**Q3**	**Q4**	***P* for trend**
**3-MONTH FOLLOW-UP**					
**Recurrence of stroke**					
Events, *n* (%)	115 (3.17)	123 (3.33)	149 (4.09)	208 (5.74)	
Crude OR (95% CI)	1	1.053 (0.813–1.363)	1.302 (1.016–1.668)	1.8f62 (1.476–2.350)	<0.0001
Adjusted OR (95% CI)	1	1.084 (0.835–1.406)	1.318 (1.026–1.693)	1.790 (1.411–2.271)	<0.0001
**Death**					
Events, *n* (%)	73 (2.01)	83 (2.24)	115 (3.14)	236 (6.42)	
Crude OR (95% CI)	1	1.121 (0.816–1.540)	1.583 (1.176–2.130)	3.353 (2.568–4.377)	<0.0001
Adjusted OR (95% CI)	1	1.343 (0.966–1.866)	1.794 (1.316–2.447)	3.249 (2.447–4.313)	<0.0001
**Poor functional outcomes**					
Events, *n* (%)	438 (12.04)	460 (12.43)	532 (14.52)	706 (19.21)	
Crude OR (95% CI)	1	1.038 (0.902–1.193)	1.241 (1.084–1.422)	1.738 (1.527–1.978)	<0.0001
Adjusted OR (95% CI)	1	1.102 (0.952–1.275)	1.289 (1.117–1.487)	1.618 (1.408–1.859)	<0.0001
**1-YEAR FOLLOW-UP**					
**Recurrence of stroke**					
Events, *n* (%)	170 (4.85)	167 (4.68)	212 (6.02)	298 (8.74)	
Crude OR (95% CI)	1	0.962 (0.773–1.197)	1.257 (1.021–1.547)	1.878 (1.546–2.281)	<0.0001
Adjusted OR (95% CI)	1	0.990 (0.793–1.236)	1.286 (1.040–1.589)	1.836 (1.501–2.245)	<0.0001
**Death**					
Events, *n* (%)	187 (5.14)	190 (5.14)	232 (6.33)	423 (11.51)	
Crude OR (95% CI)	1	0.999 (0.812–1.229)	1.248 (1.023–1.522)	2.401 (2.008–2.871)	<0.0001
Adjusted OR (95% CI)	1	1.154 (0.929–1.433)	1.405 (1.139–1.733)	2.520 (2.077–3.057)	<0.0001
**Poor functional outcomes**					
Events, *n* (%)	418 (11.49)	424 (11.46)	466 (12.72)	551 (14.99)	
Crude OR (95% CI)	1	0.997 (0.864–1.151)	1.123 (0.975–1.293)	1.359 (1.186–1.558)	<0.0001
Adjusted OR (95% CI)	1	1.067 (0.919–1.238)	1.183 (1.021–1.370)	1.301 (1.125–1.504)	0.0001

*OR, odds ratio; 95% CI, 95% confidence interval*.

*Adjustments were made for age, sex, hypertension, hyperlipidemia, diabetes, previous stroke or transient ischemic attack, myocardial infarction, atrial fibrillation, drinking, smoking and the NIHSS score at admission*.

According to the ROC curve, Table 4 shows the optimal cutoff value of leukocyte count level predicted adverse clinical outcomes and the sensitivity, the specificity, and the AUC. Leukocyte count has a moderate accuracy to predict the adverse clinical outcomes both at 3-months and at 1-year follow-up (Table 4).

**Table 4:** Receiver operating characteristic curve of leukocyte count on the adverse clinical outcomes.

	**Cutoff**	**Sensitivity**	**Specificity**	**AUC**
**3-month follow-up**				
Recurrence of stroke	1.10622	0.60000	0.50622	0.5676
Death	1.22280	0.46548	0.75732	0.6326
Poor functional outcomes	1.09380	0.33052	0.76328	0.5594
**1-year follow-up**				
Recurrence of stroke	1.11532	0.35183	0.76349	0.5707
Death	1.17157	0.40988	0.76169	0.5972
Poor functional outcomes	1.05389	0.54707	0.50683	0.5333

*AUC, area under the curve*.

### Subgroup Analysis

**Figure 1:**
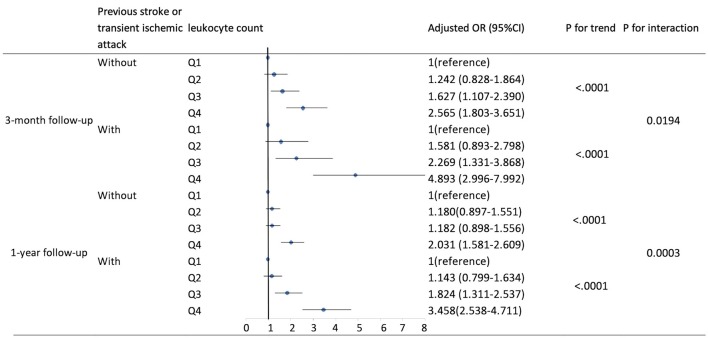
Adjusted odds ratios by leukocyte count quartiles for all-cause death at 3-months and 1-year follow-up according to history of previous stroke or transient ischemic attack. OR, odds ratio; 95% CI, 95% confidence interval. Adjustments were made for age, sex, hypertension, hyperlipidemia, diabetes, myocardial infarction, atrial fibrillation, drinking, smoking, and the NIHSS score at admission.

**Figure 2:**
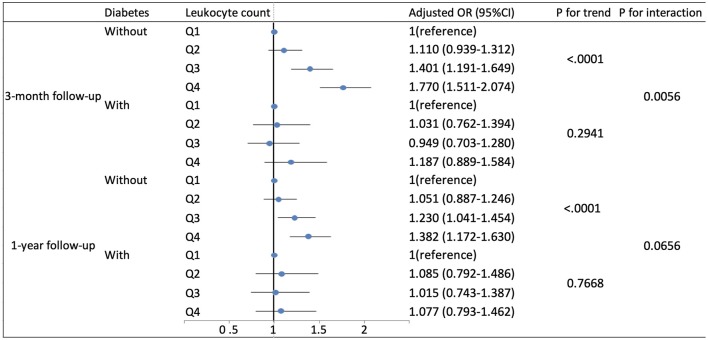
Adjusted odds ratios by leukocyte count quartiles for poor functional outcomes at 3-months and 1-year follow-up according to history of diabetes. OR, odds ratio; 95% CI, 95% confidence interval. Adjustments were made for age, sex, hypertension, hyperlipidemia, previous stroke or transient ischemic attack, myocardial infarction, atrial fibrillation, drinking, smoking, and the NIHSS score at admission.

Based on existing research results and baseline characteristics in our study, we chose age, sex, history of hypertension, history of diabetes, history of previous stroke or transient ischemic attack, and smoking status as subgroup factors. The results of subgroup analysis about the relationship between leukocyte count quartiles and risk of adverse clinical outcomes are shown in Tables 5, 6. After adjustments for age, sex, hypertension, hyperlipidemia, diabetes, previous stroke or transient ischemic attack, myocardial infarction, atrial fibrillation, drinking, smoking, and the NIHSS score at admission, in most subgroups, leukocyte count quartiles were significantly associated with adverse clinical outcomes at 3-months and 1-year follow-up. The effects of leukocyte count quartiles on adverse clinical outcomes were similar across most subgroups. However, both at 3-months and at 1-year follow-up, the effect of leukocyte count quartiles on all-cause death appeared to be more pronounced among patients who had previous stroke or transient ischemic attack (*P* = 0.0194 for the interaction at 3-months follow-up and *P* = 0.0003 for the interaction at 1-year follow-up). Moreover, at 3-months follow-up compared with the diabetes patients, those without diabetes in higher quartiles were more likely to have poor functional outcomes (*P* = 0.0056 for the interaction). Unfortunately, we did not get similar result at 1-year follow-up (*P* = 0.0656 for the interaction). In summary, there was no significant difference in the relationship between leukocyte count and adverse clinical outcomes across most subgroups such as age, sex, history of hypertension, and smoking. Meanwhile, the relationship between leukocyte count and all-cause death according to the history of previous stroke or transient ischemic attack at 3-months and 1-year follow-up was significantly different. The relationship between leukocyte count and poor functional outcomes was also significantly different in patients with and without diabetes at 3-months follow-up (Tables 5, 6; Figures 1, 2).

**Table 5:** Subgroup analysis of the relationship between leukocyte count quartiles and risk of adverse clinical outcomes at 3-month follow-up.

	**Q1**	**Q2**	**Q3**	**Q4**	***P* for trend**	***P* for interaction**
**RECURRENCE OF STROKE**						
**Age**						
<60 years						0.5605
Adjusted OR (95%CI)	1	1.306 (0.729–2.338)	1.561 (0.891–2.734)	2.090 (1.226–3.565)	0.0031	
≥60 years						
Adjusted OR (95%CI)	1	1.022 (0.763–1.369)	1.243 (0.938–1.647)	1.676 (1.282–2.189)	<0.0001	
**Sex**						
Male						0.4876
Adjusted OR (95%CI)	1	0.877 (0.622–1.237)	1.047 (0.754–1.454)	1.667 (1.227–2.264)	0.0002	
Female						
Adjusted OR (95%CI)	1	1.427 (0.956–2.131)	1.788 (1.212–2.640)	1.970 (1.349–2.877)	0.0002	
**Hypertension**						
Without						0.9570
Adjusted OR (95%CI)	1	1.378 (0.856–2.219)	1.803 (1.140–2.850)	1.948 (1.237–3.066)	0.0020	
With						
Adjusted OR (95%CI)	1	0.982 (0.719–1.340)	1.160 (0.860–1.565)	1.741 (1.316–2.304)	<0.0001	
**Previous stroke or transient ischemic attack**						
Without						0.9646
Adjusted OR (95%CI)	1	1.201 (0.848–1.701)	1.447 (1.031–2.031)	1.902 (1.374–2.632)	<0.0001	
With						
Adjusted OR (95%CI)	1	0.947 (0.639–1.403)	1.174 (0.808–1.704)	1.674 (1.178–2.378)	0.0011	
**Diabetes**						
Without						0.7228
Adjusted OR (95%CI)	1	1.092 (0.816–1.462)	1.228 (0.922–1.635)	1.890 (1.448–2.467)	<0.0001	
With						
Adjusted OR (95%CI)	1	1.068 (0.598–1.909)	1.628 (0.954–2.777)	1.558 (0.917–2.648)	0.0377	
**Smoking**						
Without						0.2723
Adjusted OR (95%CI)	1	1.352 (0.970–1.885)	1.501 (1.079–2.088)	2.072 (1.518–2.829)	<0.0001	
With						
Adjusted OR (95%CI)	1	0.765 (0.500–1.169)	1.081 (0.736–1.588)	1.435 (0.992–2.077)	0.0113	
**DEATH**						
Age						
<60 years						0.1022
Adjusted OR (95%CI)	1	3.292 (0.909–11.920)	4.901 (1.419–16.919)	8.528 (2.599–27.983)	<0.0001	
≥60 y						
Adjusted OR (95%CI)	1	1.192 (0.845–1.682)	1.558 (1.127–2.154)	2.730 (2.035–3.662)	<0.0001	
**Sex**						
Male						0.9391
Adjusted OR (95%CI)	1	1.447 (0.920–2.277)	1.608 (1.033–2.506)	3.513 (2.356–5.238)	<0.0001	
Female						
Adjusted OR (95%CI)	1	1.195 (0.736–1.940)	1.986 (1.281–3.080)	3.091 (2.062–4.633)	<0.0001	
**Hypertension**						
Without						0.2086
Adjusted OR (95%CI)	1	1.264 (0.766–2.083)	1.845 (1.158–2.942)	2.741 (1.780–4.221)	<0.0001	
With						
Adjusted OR (95%CI)	1	1.420 (0.913–2.208)	1.818 (1.197–2.762)	3.651 (2.494–5.346)	<0.0001	
**Previous stroke or transient ischemic attack**						
Without						0.0194
Adjusted OR (95%CI)	1	1.242 (0.828–1.864)	1.627 (1.107–2.390)	2.565 (1.803–3.651)	<0.0001	
With						
Adjusted OR (95%CI)	1	1.581 (0.893–2.798)	2.269 (1.331–3.868)	4.893 (2.996–7.992)	<0.0001	
**Diabetes**						
Without						0.4456
Adjusted OR(95%CI)	1	1.380 (0.959–1.986)	1.674 (1.179–2.376)	3.253 (2.372–4.462)	<0.0001	
With						
Adjusted OR(95%CI)	1	1.246 (0.571–2.719)	2.327 (1.158–4.677)	3.523 (1.824–6.804)	<0.0001	
**Smoking**						
Without						0.1505
Adjusted OR(95%CI)	1	1.661 (1.103–2.502)	2.113 (1.428–3.128)	3.894 (2.722–5.571)	<0.0001	
With						
Adjusted OR(95%CI)	1	0.888 (0.505–1.562)	1.345 (0.808–2.239)	2.421 (1.517–3.863)	<0.0001	
**POOR FUNCTIONAL OUTCOMES**						
**Age**						
<60 years						0.1260
Adjusted OR (95%CI)	1	1.100 (0.773–1.564)	1.173 (0.830–1.657)	1.749 (1.270–2.409)	0.0001	
≥60 years						
Adjusted OR (95%CI)	1	1.073 (0.914–1.259)	1.286 (1.100–1.504)	1.497 (1.284–1.747)	<0.0001	
**Sex**						
Male						0.1519
Adjusted OR (95%CI)	1	1.127 (0.925–1.374)	1.339 (1.106–1.622)	1.731 (1.436–2.088)	<0.0001	
Female						
Adjusted OR (95%CI)	1	1.082 (0.870–1.345)	1.230 (0.990–1.528)	1.471 (1.193–1.813)	0.0001	
**Hypertension**						
Without						0.7175
Adjusted OR (95%CI)	1	0.979 (0.760–1.261)	1.144 (0.892–1.468)	1.552 (1.224–1.969)	0.0001	
With						
Adjusted OR (95%CI)	1	1.173 (0.980–1.404)	1.371 (1.151–1.635)	1.672 (1.408–1.985)	<0.0001	
**Previous stroke or transient ischemic attack**						
Without						0.3029
Adjusted OR (95%CI)	1	1.034 (0.854–1.253)	1.223 (1.014–1.475)	1.663 (1.390–1.990)	<0.0001	
With						
Adjusted OR(95%CI)	1	1.207 (0.962–1.514)	1.394 (1.116–1.740)	1.571 (1.262–1.956)	<0.0001	
**Diabetes**						
Without						0.0056
Adjusted OR(95%CI)	1	1.110 (0.939–1.312)	1.401 (1.191–1.649)	1.770 (1.511–2.074)	<0.0001	
With						
Adjusted OR(95%CI)	1	1.031 (0.762–1.394)	0.949 (0.703–1.280)	1.187 (0.889–1.584)	0.2941	
**Smoking**						
Without						0.4488
Adjusted OR(95%CI)	1	1.172 (0.978–1.405)	1.279 (1.067–1.533)	1.615 (1.355–1.925)	<0.0001	
With						
Adjusted OR(95%CI)	1	0.984 (0.767–1.262)	1.294 (1.024–1.635)	1.618 (1.287–2.033)	<0.0001	

*OR, odds ratio; 95%CI, 95% confidence interval*.

*Adjustments included age, sex, hypertension, hyperlipidemia, diabetes, previous stroke, or transient ischemic attack, myocardial infarction, atrial fibrillation, drinking, smoking and the NIHSS score at admission*.

**Table 6:** Subgroup analysis of the relationship between leukocyte count quartiles and risk of adverse clinical outcomes at 1-year follow-up.

	**Q1**	**Q2**	**Q3**	**Q4**	***P* for trend**	***P* for interaction**
**RECURRENCE OF STROKE**						
**Age**						
<60 years						0.7320
Adjusted OR (95%CI)	1	1.166 (0.697–1.951)	1.508 (0.926–2.456)	2.012 (1.265–3.200)	0.0008	
≥60 years						
Adjusted OR (95%CI)	1	0.938 (0.733–1.200)	1.205 (0.952–1.525)	1.727 (1.381–2.160)	<0.0001	
**Sex**						
Male						0.9437
Adjusted OR (95%CI)	1	0.921 (0.683–1.241)	1.185 (0.894–1.570)	1.866 (1.428–2.440)	<0.0001	
Female						
Adjusted OR (95%CI)	1	1.076 (0.771–1.500)	1.426 (1.034–1.966)	1.814 (1.336–2.463)	<0.0001	
**Hypertension**						
Without						0.7603
Adjusted OR (95%CI)	1	1.622 (1.062–2.478)	1.896 (1.248–2.881)	2.256 (1.496–3.402)	<0.0001	
With						
Adjusted OR (95%CI)	1	0.822 (0.632–1.069)	1.122 (0.877–1.436)	1.719 (1.364–2.167)	<0.0001	
**Previous stroke or transient ischemic attack**						
Without						0.1075
Adjusted OR (95%CI)	1	1.089 (0.806–1.471)	1.241 (0.923–1.668)	1.691 (1.274–2.244)	0.0001	
With						
Adjusted OR (95%CI)	1	0.883 (0.635–1.227)	1.329 (0.981–1.802)	2.013 (1.509–2.686)	<0.0001	
**Diabetes**						
Without						0.8801
Adjusted OR (95%CI)	1	1.019 (0.790–1.313)	1.323 (1.038–1.687)	1.870 (1.483–2.359)	<0.0001	
With						
Adjusted OR (95%CI)	1	0.909 (0.573–1.443)	1.195 (0.774–1.845)	1.754 (1.164–2.643)	0.0009	
**Smoking**						
Without						0.5295
Adjusted OR (95%CI)	1	1.129 (0.855–1.492)	1.419 (1.082–1.862)	1.991 (1.539–2.576)	<0.0001	
With						
Adjusted OR (95%CI)	1	0.795 (0.549–1.150)	1.090 (0.776–1.531)	1.642 (1.189–2.269)	0.0002	
**DEATH**						
**Age**						
<60 years						0.2641
Adjusted OR (95%CI)	1	1.442 (0.716–2.903)	1.958 (1.008–3.801)	3.131 (1.688–5.809)	<0.0001	
≥60 years						
Adjusted OR (95%CI)	1	1.065 (0.850–1.335)	1.262 (1.014–1.569)	2.189 (1.792–2.674)	<0.0001	
**Sex**						
Male						0.8473
Adjusted OR (95%CI)	1	1.230 (0.922–1.642)	1.257 (0.943–1.676)	2.716 (2.090–3.529)	<0.0001	
Female						
Adjusted OR (95%CI)	1	1.045 (0.750–1.455)	1.599 (1.175–2.176)	2.360 (1.771–3.144)	<0.0001	
**Hypertension**						
Without						0.4873
Adjusted OR (95%CI)	1	1.226 (0.862–1.744)	1.401 (0.989–1.983)	2.471 (1.797–3.398)	<0.0001	
With						
Adjusted OR (95%CI)	1	1.126 (0.854–1.483)	1.416 (1.088–1.843)	2.559 (2.005–3.266)	<0.0001	
**Previous stroke or transient ischemic attack**						
Without						0.0003
Adjusted OR (95%CI)	1	1.180 (0.897–1.551)	1.182 (0.898–1.556)	2.031 (1.581–2.609)	<0.0001	
With						
Adjusted OR (95%CI)	1	1.143 (0.799–1.634)	1.824 (1.311–2.537)	3.458 (2.538–4.711)	<0.0001	
**Diabetes**						
Without						0.2879
Adjusted OR (95%CI)	1	1.214 (0.953–1.546)	1.450 (1.145–1.836)	2.518 (2.024–3.134)	<0.0001	
With						
Adjusted OR (95%CI)	1	0.960 (0.586–1.572)	1.295 (0.817–2.054)	2.530 (1.660–3.856)	<0.0001	
**Smoking**						
Without						0.9006
Adjusted OR (95%CI)	1	1.270 (0.976–1.654)	1.572 (1.217–2.031)	2.516 (1.981–3.196)	<0.0001	
With						
Adjusted OR (95%CI)	1	0.934 (0.636–1.371)	1.112 (0.771–1.604)	2.549 (1.836–3.539)	<0.0001	
**POOR FUNCTIONAL OUTCOMES**						
**Age**						
<60 years						0.1330
Adjusted OR (95%CI)	1	1.053 (0.715–1.552)	1.052 (0.717–1.545)	1.477 (1.035–2.107)	0.0192	
≥60 years						
Adjusted OR (95%CI)	1	1.044 (0.889–1.226)	1.187 (1.013–1.390)	1.211 (1.034–1.419)	0.0062	
**Sex**						
Male						0.8761
Adjusted OR (95%CI)	1	0.963 (0.787–1.178)	1.142 (0.939–1.388)	1.232 (1.013–1.497)	0.0103	
Female						
Adjusted OR (95%CI)	1	1.210 (0.971–1.509)	1.235 (0.987–1.544)	1.397 (1.125–1.736)	0.0034	
**Hypertension**						
Without						0.4193
Adjusted OR (95%CI)	1	0.903 (0.695–1.174)	1.055 (0.816–1.366)	1.156 (0.897–1.489)	0.1734	
With						
Adjusted OR (95%CI)	1	1.155 (0.963–1.386)	1.254 (1.047–1.501)	1.393 (1.166–1.664)	0.0002	
**previous stroke or transient ischemic attack**						
Without						0.1956
Adjusted OR(95%CI)	1	0.974 (0.798–1.189)	1.161 (0.956–1.411)	1.357 (1.122–1.640)	0.0004	
With						
Adjusted OR(95%CI)	1	1.201 (0.958–1.505)	1.220 (0.974–1.528)	1.244 (0.994–1.557)	0.0702	
**Diabetes**						
Without						0.0656
Adjusted OR(95%CI)	1	1.051 (0.887–1.246)	1.230 (1.041–1.454)	1.382 (1.172–1.630)	<0.0001	
With						
Adjusted OR(95%CI)	1	1.085 (0.792–1.486)	1.015 (0.743–1.387)	1.077 (0.793–1.462)	0.7668	
**Smoking**						
Without						0.5447
Adjusted OR(95%CI)	1	1.174 (0.977–1.411)	1.193 (0.990–1.437)	1.420 (1.184–1.702)	0.0003	
With						
Adjusted OR(95%CI)	1	0.879 (0.681–1.135)	1.147 (0.902–1.457)	1.129 (0.887–1.437)	0.0984	

*OR, odds ratio; 95%CI, 95% confidence interval*.

*Adjustments included age, sex, hypertension, hyperlipidemia, diabetes, previous stroke or transient ischemic attack, myocardial infarction, atrial fibrillation, drinking, smoking and the NIHSS score at admission*.

## Discussion

In this analysis of the CSNR II study, we firstly validated that in acute ischemic stroke patients, higher leukocyte count within the first 24 h after admission was associated with short-term and long-term adverse clinical outcomes, including recurrent stroke, all-cause death, and poor functional outcomes. According to Table 1, the patients we excluded had a higher proportion of risk factors of ischemic stroke and a higher severity of stroke. Therefore, they might be more likely to have adverse clinical outcomes, and this might influence the predictive value of leukocyte count for adverse clinical outcomes in our study. Besides, we found that although there was no significant difference in the relationship between leukocyte count and adverse clinical outcomes according to age, sex, history of hypertension and smoking, the effect of leukocyte count on both short-term and long-term all-cause death was more pronounced among patients with previous stroke or transient ischemic attack, and a similar result was found in the effect of leukocyte count on only short-term poor functional outcomes among patients without diabetes. These results may suggest that leukocyte count at admission after acute cerebrovascular events was useful in estimating clinical outcomes, especially in patients with certain specific characteristics.

After a stroke onset, adrenal gland would release stress hormones, particularly catecholamines immediately and trigger robust activation of the immune system (2, 3, 15) via the lymphoid organs innervated by sympathetic directly (16, 17). At the same time, necrotic cell in the ischemic area could cause the release of proinflammatory cytokines, chemokines, and the recruitment of immune cells into the central nervous system (18, 19). Overall, post-ischemic brain tissue is manifested as endothelial activation, barrier dysfunction, enhanced generation of inflammatory mediators, and recruitment of leukocytes (20). Among them, the impact of leukocyte recruitment is a controversial issue (18). Those cells might participate in tissue repair, but their detrimental effects are more significant (18). Previous studies have suggested that increased numbers and activation of circulating leukocytes could actively contribute to organ ischemia by increased adhesion to and damage of the endothelium (21). Leukocytes could liberate proteases, inflammatory mediators, and free radicals that cause proteolytic and oxidative damage to endothelial cells (8). Besides, leukocytes are larger than other blood cells and may plug the microvasculature directly (22). In summary, increased and activated leukocytes are involved in pathophysiology of cerebral ischemic injury through various mechanisms and influence its development. Thus, leukocytes are thought to be able to predict and affect the clinical outcomes following acute ischemic stroke (18).

Recently, several animal experiments suggest certain characteristic differences in post-ischemic inflammatory responses that may contribute to clinical outcomes. Exacerbated inflammatory responses have been observed in old, diabetic, and hypertensive mice after ischemic stroke, which lead to more severe brain damage and adverse clinical outcomes. However, little is known about the immunological mechanisms underlying the above effects. The possible explanation of age differences is reported to be that aging is characterized by a chronic low-grade inflammatory state with an increase of systemic inflammatory cytokines (10, 23–25). The contributors of sex differences might be the different endocrine states, sex-specific genetic coding, epigenetic control of gene expression, and sex-specific changes in microbiome, metabolism, and coagulation (11, 26, 27). As for the effect of diabetes, the explanation is that diabetes involves chronic systemic low-grade inflammation manifested by reactive oxygen species generation, proinflammatory cytokines expression, and other inflammatory mediator activation (28). The finding surrounding hypertension could be explained by increased numbers and activation status of circulating myeloid leukocytes and increased levels of leukocyte-attracting chemokines (13).

However, we did not get similar results with previous animal experiments. We found that there was no significant difference in the relationship between leukocyte count and adverse clinical outcomes according to age, sex, history of hypertension, and smoking. We think that the reasons for the inconsistency of the results in patient analysis and animal experiments may be as follows. Firstly, the inflammatory response after acute ischemic stroke is a complicated process, and the effects of different factors on post-ischemic inflammatory response would be more complicated. The mechanisms and targets of those factors remain unknown. So, only choosing leukocyte count might be inappropriate. Those factors might influence inflammatory response not only through the recruitment, increased numbers, or activation of leukocytes. Secondly, previous animal experiments mostly used a mouse model of acute ischemia stroke. The effects of factors on post-ischemic inflammatory response might be different between mice and patients. Thus, the relationship between inflammatory response and adverse clinical outcomes in subgroups might be also different between mice and patients.

Moreover, we found that it was significantly different in the relationship between leukocyte count and all-cause death according to history of previous stroke or transient ischemic attack. The most plausible explanation is that in patients with previous stroke or transient ischemic attack, there could have been chronic low-grade inflammatory state in the central nervous system, and when ischemic stroke occurs again, the inflammatory response might be significantly enhanced and rapidly activated, thus affecting the clinical outcomes. Although diabetes is thought to involve chronic systemic low-grade inflammation, we found the association of higher leukocyte count with poorer 3-months functional outcomes in acute ischemic stroke patients without diabetes, and the reasons for this remain unknown. The possible explanation is that the inflammatory response after ischemic stroke is different in the patients with and without diabetes. Maybe the post-ischemic inflammatory response in patients with diabetes is characterized by inflammatory mediator activation and does not involve a significant increase in leukocyte count.

The main strength of our study is the large, multicentric, consecutive patient inclusion design and prospective collection of demographic, clinical, follow-up data. However, there were still some limitations in our study. Firstly, equipment heterogeneity at research centers may lead to biased estimates of results, but this may have little impact, because the equipment in every research center is under strict quality control in daily use. Secondly, our study only evaluated leukocyte count. Other inflammatory markers might be more significant in reflecting the effect of different factors on inflammatory response after acute ischemic stroke. Thirdly, our study did not have dynamic data about leukocyte count and did not evaluate the change of leukocyte count during hospitalization. However, the dynamic change of leukocyte count over time may provide valuable information to understand the underlying mechanism of inflammatory response after stroke and its clinical significance. Fourthly, the median (IQR) of the NIHSS score at admission of total patients included in our analysis was 4 (2–6). The severity of ischemic stroke might be mild and this could limit the ability to estimate the association of leukocyte count with clinical outcomes in more severe ischemic stroke patients. Finally, residual bias may still exist because of the influence of comorbidities or environmental factors such as tumor, trauma, and acute toxicosis.

## Conclusion

Our results suggest that leukocyte count at admission is associated with short-term and long-term clinical outcomes in acute ischemic stroke patients and may have a role as a predictor for clinical outcomes, especially in patients with certain specific characteristics, although it needs further research to confirm that.

## Data Availability Statement

All datasets generated for this study are included in the article/supplementary material.

## Ethics Statement

The studies involving human participants were reviewed and approved by Central Institutional Review Board of the Beijing Tiantan Hospital. The patients/participants provided their written informed consent to participate in this study.

## Author Contributions

KQ and YW conceptualized this work. Statistical analysis was undertaken by AW, XZ, and KQ. KQ prepared the manuscript. YW was the guarantor of this paper. All authors approved the protocol.

### Conflict of Interest

The authors declare that the research was conducted in the absence of any commercial or financial relationships that could be construed as a potential conflict of interest.

## References

[1] Zhao L Dai Q Chen X Li S Shi R Yu S. Neutrophil-to-lymphocyte ratio predicts length of stay and acute hospital cost in patients with acute ischemic stroke. J Stroke Cerebrovas Dis. (2016) 25:739–44. 10.1016/j.jstrokecerebrovasdis.2015.11.012 26775271

[2] Macrez R Ali C Toutirais O Le Mauff B Defer G Dirnagl U. Stroke and the immune system: from pathophysiology to new therapeutic strategies. Lancet Neurol. (2011) 10:471–80. 10.1016/S1474-4422(11)70066-7 21511199

[3] Chamorro A Meisel A Planas AM Urra X van de Beek D Veltkamp R. The immunology of acute stroke. Nat. Rev. Neurol. (2012) 8:401–10. 10.1038/nrneurol.2012.98 22664787

[4] Welsh P Barber M Langhorne P Rumley A Lowe GD Stott DJ. Associations of inflammatory and haemostatic biomarkers with poor outcome in acute ischaemic stroke. Cerebrovasc Dis. (2009) 27:247–53. 10.1159/000196823 19176958

[5] Nardi K Milia P Eusebi P Paciaroni M Caso V Agnelli G. Admission leukocytosis in acute cerebral ischemia: influence on early outcome. J Stroke Cerebrovasc Dis. (2012) 21:819–24. 10.1016/j.jstrokecerebrovasdis.2011.04.015 21703875

[6] Buck BH Liebeskind DS Saver JL Bang OY Yun SW Starkman S. Early neutrophilia is associated with volume of ischemic tissue in acute stroke. Stroke. (2008) 39:355–60. 10.1161/STROKEAHA.107.490128 18162626

[7] Whiteley W Chong WL Sengupta A Sandercock P. Blood markers for the prognosis of ischemic stroke: a systematic review. Stroke. (2009) 40:e380–9. 10.1161/STROKEAHA.108.528752 19286602

[8] Grau AJ Boddy AW Dukovic DA Buggle F Lichy C Brandt T. Leukocyte count as an independent predictor of recurrent ischemic events. Stroke. (2004) 35:1147–52. 10.1161/01.STR.0000124122.71702.64 15017013

[9] Ritzel RM Lai YJ Crapser JD Patel AR Schrecengost A Grenier JM. Aging alters the immunological response to ischemic stroke. Acta Neuropathol. (2018) 136:89–110. 10.1007/s00401-018-1859-2 29752550 PMC6015099

[10] Shen F Jiang L Han F Degos V Chen S Su H. Increased inflammatory response in old mice is associated with more severe neuronal injury at the acute stage of ischemic stroke. Aging Dis. (2019) 10:12–22. 10.14336/AD.2018.0205 30705764 PMC6345332

[11] Spychala MS Honarpisheh P McCullough LD Sex differences in neuroinflammation and neuroprotection after ischemic stroke. J Neurosci Res. (2017) 95:462–71. 10.1002/jnr.23962 27870410 PMC5217708

[12] Kim E Tolhurst AT Cho S. Deregulation of inflammatory response in the diabetic condition is associated with increased ischemic brain injury. J Neuroinflam. (2014) 11:83. 10.1186/1742-2094-11-83 24886035 PMC4017808

[13] Möller K Pösel C Kranz A Schulz I Scheibe J Didwischus N. Arterial hypertension aggravates innate immune responses after experimental stroke. Front Cell Neurosci. (2015) 9:461. 10.3389/fncel.2015.00461 26640428 PMC4661280

[14] Li Z Wang C Zhao X Liu L Wang C Li H China national stroke registries. Substantial progress yet significant opportunity for improvement in stroke care in China. Stroke. (2016) 47:2843–9. 10.1161/STROKEAHA.116.014143 27758941

[15] Iadecola C Anrather J. The immunology of stroke: from mechanisms to translation. Nat Med. (2011) 17:796–808. 10.1038/nm.2399 21738161 PMC3137275

[16] Vogelgesang A Becker KJ Dressel A. Immunological consequences of ischemic stroke. Acta Neurol Scand. (2014) 129:1–12. 10.1111/ane.12165 23848237

[17] Winklewski PJ Radkowski M Demkow U. Cross-talk between the inflammatory response, sympathetic activation and pulmonary infection in the ischemic stroke. J Neuroinflam. (2014) 11:213. 10.1186/s12974-014-0213-4 25539803 PMC4297381

[18] Ruhnau J Schulze J Dressel A Vogelgesang A. Thrombosis, neuroinflammation, and poststroke infection: the multifaceted role of neutrophils in stroke. J Immunol Res. (2017) 2017:5140679. 10.1155/2017/5140679 28331857 PMC5346374

[19] Worthmann H Tryc AB Deb M Goldbecker A Ma YT Tountopoulou A Linking infection and inflammation in acute ischemic stroke. Ann N Y Acad Sci. (2010) 12076:116–22. 10.1111/j.1749-6632.2010.05738.x 20955434

[20] Ishikawa M Zhang JH Nanda A Granger DN. Inflammatory responses to ischemia and reperfusion in the cerebral microcirculation. Front Biosci. (2004) 9:1339–47. 10.2741/1330 14977549

[21] Mazzoni MC Schmid-Schonbein GW. Mechanisms and consequences of cell activation in the microcirculation. Cardiovasc Res. (1996) 32:709–19. 10.1016/S0008-6363(96)00146-0 8915189

[22] Rasouli M Nesarhosseini V Kiasari AM Arab S Shariati R Kazemi D. The multiplicative interactions of leukocyte counts with some other risk factors enhance the prognostic value for coronary artery disease. Cardiol J. (2011) 18:246–53. 21660913

[23] Jiang T Cadenas E. Astrocytic metabolic and inflammatory changes as a function of age. Aging Cell. (2014) 13:1059–1067. 10.1111/acel.12268 25233945 PMC4244278

[24] Franceschi C Campisi J. Chronic inflammation (inflammaging) and its potential contribution to age-associated diseases. J Gerontol A Biol Sci Med Sci. (2014) 69:S4–9. 10.1093/erona/glu057 24833586

[25] Popa-Wagner A Buga AM Kokaia Z. Perturbed cellular response to brain injury during aging. Ageing Res Rev. (2011) 10:71–9. 10.1016/j.arr.2009.10.008 19900590

[26] Maney DL. Perils and pitfalls of reporting sex differences. Philos Trans R Soc Lond B Biol Sci. (2016) 371:20150119. 10.1098/rstb.2015.0119 26833839 PMC4785904

[27] Roy-O'Reilly M McCullough LD. Sex differences in stroke: the contribution of coagulation. Exp Neurol. (2014) 259:16–27. 10.1016/j.expneurol.2014.02.011 24560819 PMC4127336

[28] Shukla V Shakya AK Perez-Pinzon MA Dave KR. Cerebral ischemic damage in diabetes: an inflammatory perspective. J Neuroinflamm. (2017) 14:21 10.1186/s12974-016-0774-5 28115020 PMC5260103

